# Histological study of the role of CD34+ stem cells and mast cells in cyclophosphamide-induced thymic injury in rats and the possible attenuating role of melatonin

**DOI:** 10.1007/s00418-023-02185-6

**Published:** 2023-03-08

**Authors:** Amira I. Shrief, Walaa H. E. Hamed, Shireen A. Mazroa, Amal M. Moustafa

**Affiliations:** Department of Medical Histology and Cell Biology, Faculty of Medicine, Mansoura University, Mansoura, Egypt

**Keywords:** Thymus gland, Cyclophosphamide, Melatonin, CD34+ stem cells, Mast cells

## Abstract

Cyclophosphamide (CP) is an anticancer drug that adversely affects immunity and thymus structure. Melatonin is a hormone secreted by the pineal gland. It boosts immunity and has antioxidant properties. Therefore, the present study was conducted to investigate the possible protective effect of melatonin on CP-induced changes in the rat thymus. Forty male albino rats were used and divided equally into four main groups. Group I was the control group. Group II (melatonin group) received melatonin at a dose of 10 mg/kg body weight/day by intraperitoneal injection throughout the experimental period. Group III (CP group) received 200 mg/kg body weight CP by a single intraperitoneal injection. Group IV (CP + melatonin group) received melatonin intraperitoneally at a dose of 10 mg/kg body weight/day starting 5 days prior to CP injection until the end of the experiment. All rats were euthanized 7 days after CP injection. Administration of CP in group III resulted in depletion of the cortical thymoblasts. In addition, CD34-immunopositive stained stem cells decreased and mast cell infiltration increased. Electron microscopy showed degeneration of thymoblasts and vacuolization of epithelial reticular cells. Administration of melatonin with CP in group IV showed considerable protection of thymic histology. In conclusion, melatonin may protect against CP-induced thymic injury.

**Supplementary Information:**

The online version contains supplementary material available at 10.1007/s00418-023-02185-6.

## Introduction

Cyclophosphamide (CP) is one of the oldest anticancer drugs. It was discovered in 1958 and introduced for cancer treatment in 1959. It has been used to treat several human tumors, including breast cancer, ovarian cancer, small cell lung cancer, lymphoma, and leukemia (Braunstein et al. 2022; Hasanah et al. 2021). Because it is a potent immunosuppressant, it is commonly used for bone marrow transplantation in hematopoietic malignancies and aplastic anemia (Andolina et al. 2022).

Activation of CP by the hepatic cytochrome P-450 enzyme system results in reactive metabolites. These metabolites promote chemical alkylation of proteins, generate DNA crosslinks, trigger the generation of a wide range of reactive oxygen species (ROS), and cause cytotoxic effects (Rachmale et al. 2022). It has been reported that the immunotoxic effects of cyclophosphamide can induce thymic apoptosis, hypocellularity ,and atrophy (Senin et al. 2018).

Melatonin is the main secretion of the pineal gland. It has multiple physiological functions, including regulation of circadian rhythms, sexual behavior, and enhancement of immunity (Najafi et al. 2019). It can also be used as a protection for pathological conditions such as sleep disorders, Alzheimer's disease, stroke, depression, migraines, convulsions, and cancer (Goudarzi et al. 2017). Melatonin has been proven to have anti-inflammatory and strong antioxidant properties (Hagström et al. 2022). It has also been reported that melatonin plays an important role in the development of T cells in the thymus. It enhances the proliferative activity and the rate of DNA synthesis in lymphocytes and partially prevents age-related thymic involution. Furthermore, treatment with melatonin reduces the rate of apoptosis when the thymus is exposed to various toxic substances (Ren et al. 2017). A previous study also reported that melatonin has a protective effect against CP-induced lung injury (Shokrzadeh et al. 2015).

CD34 is a transmembrane phosphorylated glycoprotein. It is used as a surface marker to identify bone marrow-derived multipotent stem cells that have the capacity to differentiate into T cells in the thymus gland. CD34+ stem cells play a main role in thymus regeneration (Chowdhury and Ghosh 2021).

Thymic mast cells are usually found in the connective tissue of the capsule and interlobular septa, inside the thymic lobules and mainly in the perivascular spaces (Ribatti 2019). The number and size of mast cells increase in aging and cases of immunodeficiency (Polevshchikov and Guselnikova 2021). Mast cells have important immunological functions. They are able to synthesize and release many growth factors and cytokines that stimulate thymocyte and thymic epithelial cell function (Ribatti 2019).

This work was conducted to study the histological changes in the rat thymus caused by CP and to assess the possible protective effects of melatonin.

## Materials and methods

### Chemicals

Cyclophosphamide (Endoxan^®^) (200 mg/vial dry powder) was purchased from Baxter Oncology GMBH (Westfalen, Germany). Melatonin (5 mg/tablet) was purchased from Puritan's Pride, USA.

### Animals and experimental design

All rat experimental procedures and protocols were performed in accordance with international guidelines for the use of laboratory animals, and approval was obtained from the Mansoura Faculty of Medicine Institutional Research Board (MFM-IRB; approval code number MD.19.02.138).

Forty male albino rats were used and were divided equally into four main groups. Group I (control group) included 10 rats that received normal saline at a dose of 0.5 ml/day by intraperitoneal injection throughout the experimental period. Group II (melatonin-treated group) included 10 rats that received melatonin at a dose of 10 mg/kg body weight/day (Haghi-Aminjan et al. 2018) by intraperitoneal injection throughout the experimental period. Group III (CP-treated group) included 10 rats that received 200 mg/kg body weight CP (Zhai et al. 2018) by a single intraperitoneal injection. Group IV (CP + melatonin-treated group) included 10 rats that received melatonin at a dose of 10 mg/kg body weight/day (Haghi-Aminjan et al. 2018) by intraperitoneal injection starting 5 days before CP injection until the end of the experiment. All rats were euthanized 7 days after CP injection.

### Obtaining the specimens

At the end of the experiment, rats were anesthetized intraperitoneally with sodium pentobarbital 40 mg/kg (Ma et al. 2009). The thymus gland was rapidly dissected and processed to prepare paraffin sections that were used in histological and immunohistochemical studies. Paraffin sections were stained with hematoxylin and eosin (H&E) and anti-CD34 immunohistochemical staining. Other thymus specimens, approximately 1 mm in size, were fixed in a mixture of glutaraldehyde (2.5%) and paraformaldehyde (2.5%), postfixed in osmium tetroxide (1%), dehydrated, and embedded in resin to obtain semi-thin sections which were stained with toluidine blue and ultrathin sections to be examined by transmission electron microscopy.

### Light microscopy examination

Thymus gland samples were fixed in 10% buffered neutral formalin for 24 h and processed using the standard procedure to prepare paraffin blocks. Serial sections approximately 5 µm thick were cut and stained with H&E for routine histological examination of the thymic structural changes according to Suvarna et al. (2013).

### Immunohistochemical staining

Immunohistochemical studies performed anti-CD34 staining to detect hematopoietic stem cells. The primary antibody against CD34 was a mouse monoclonal antibody of the immunoglobulin G1 (IgG1) class (anti-CD34, catalogue number: M 2165, Dako, Carpinteria, CA, USA; dilution 1:40; by manufacturer). Serial paraffin sections 4–5 um thick were deparaffinized and rehydrated. The endogenous peroxidase activity was blocked with 0.3% hydrogen peroxide in absolute alcohol for 10 min. The slides were washed for 10 min in phosphate-buffered saline (PBS) at pH 7.4. To unmask the antigenic sites, sections were placed in citrate buffer (pH 6) in a microwave for 10 min. Slides were incubated in 1% bovine serum albumin (BSA) dissolved in PBS for 20 min at 37 °C in order to prevent nonspecific background staining. Slides were incubated with the primary antibody for 1.5 h at room temperature, washed, incubated with the secondary (biotinylated goat anti-polyvalent) antibody for 10 min at room temperature in a humidified chamber, and then incubated with avidin–biotin complex. Finally, sections were developed with 0.05% 3,3′-diaminobenzidine for 10 min. Slides were counterstained with Mayer’s hematoxylin, dehydrated, cleared in xylene, mounted by DPX, and covered with glass coverslips (Suvarna et al. 2013). After omitting the primary antibody, negative control thymus sections were placed under the same conditions. Additionally, CD34-stained myocardial sections were used as a positive control (Varga et al. 2016) to assess the positivity of the tissue sections. Several studies indicate the specificity of CD34 antibodies to cardiac stem cells (Nour et al. 2017; Hasanin et al. 2022).

### Electron microscopy examination

A small sample of thymus, approximately 1 mm^3^, was fixed in 2.5% glutaraldehyde and 2.5% paraformaldehyde in 0.1 M phosphate buffer (pH 7.4) overnight and then in 1% osmium tetroxide at 4 °C, postfixed, dehydrated, clarified, infiltrated in epoxy resin, and polymerized at 60 °C for 24 h. Samples were then prepared to obtain semi-thin sections (1 μm) stained with toluidine blue and ultrathin sections (60 nm) stained with uranyl acetate for 10–15 min and lead citrate for 7 min, which were then examined using a JEOL transmission electron microscope (100 CX; Tokyo, Japan) at the Faculty of Science, Alexandria University, Alexandria, Egypt.

### Image acquisition

Images for light microscopy were taken with a ToupCam digital camera (XCAM1080PHA; 2.8 pixels, UK) using ToupView software (11/4/19728) connected to an Olympus^®^ microscope (CX23LEDRF; Japan) using ×10, ×40, and ×100 oil plan achromat objective lenses with NA = 0.25, 0.65, and 1.25, respectively).

### Computer-assisted digital image analysis

Six non-overlapping randomly selected fields from each animal in each group were photographed using the objective lens for the required analysis. The resulting images were analyzed using the Photoshop CS6 and ImageJ programs with special built-in routines for automatic object analysis. They were used to assess the following:

1. The number of cortical thymoblasts/field (×1000) in H&E-stained sections: Using Photoshop CS6, the original image was converted into a black-and-white scale image. All other color components in the image were eliminated, leaving only the black shades of thymoblasts. The image was processed using ImageJ and converted to a threshold image using the steps (Image > Adjust > Threshold) in the program's menu, and the threshold was adjusted accordingly. Thymoblasts were selected and counted using the multipoint tool icon.
2. The number of cortical CD34 positively stained stem cells/field (×400) in immunohistochemically stained sections: Using Photoshop CS6, the original image was converted into a black-and-white scale image. All other color components in the image were eliminated, leaving only the black shades of brown immunostained cells. The image was processed using ImageJ and converted into a threshold image using the steps (Image > Adjust > Threshold) in the program's menu, and the threshold was adjusted accordingly. Stem cells were selected and counted using the multipoint tool icon.
3. The number of mast cells/field (×400) in semi-thin sections stained with toluidine blue: Using Photoshop CS6, the original image was converted into a black-and-white scale image. All other color components in the image were eliminated, leaving only the black shades of the purple stained mast cells. The image was processed using ImageJ and converted into a threshold image using the steps (Image > Adjust > Threshold) in the program's menu, and the threshold was adjusted accordingly. Mast cells were selected and counted with the multipoint tool icon.

Six non-overlapping, randomly selected fields of the thymic cortex at ×2000 magnification from each animal in all groups were photographed using a JEOL transmission electron microscope (100 CX; Tokyo, Japan). They were used to determine the number of cytoplasmic vacuoles/fields in the epithelial reticular cells (ERCs) (×2000). The original images were analyzed using the ImageJ program and converted into the threshold image using the steps (Image > Adjust > Threshold) in the program's menu, and the threshold was adjusted as needed. Cytoplasmic vacuoles were selected and counted with the multipoint tool icon.

### Statistical study

Morphometric data were tabulated, coded, and analyzed using the SPSS (Statistical Package for Social Sciences) version 20 computer program (IBM Corp., Armonk, NY, USA). Descriptive statistics were calculated in terms of mean ± standard deviation (± SD). For comparison between the different groups, the significance of difference was tested using one-way analysis of variance (ANOVA) for comparison among more than two groups of parametric (numerical) data, followed by post hoc Tukey for multiple comparisons. A *P* value < 0.05 was considered statistically significant.

## Results

Regarding rat mortality, two rats in the CP-treated group died during the experiment.

### Light microscopy results

#### Hematoxylin and eosin-stained sections

**Fig. 1:**
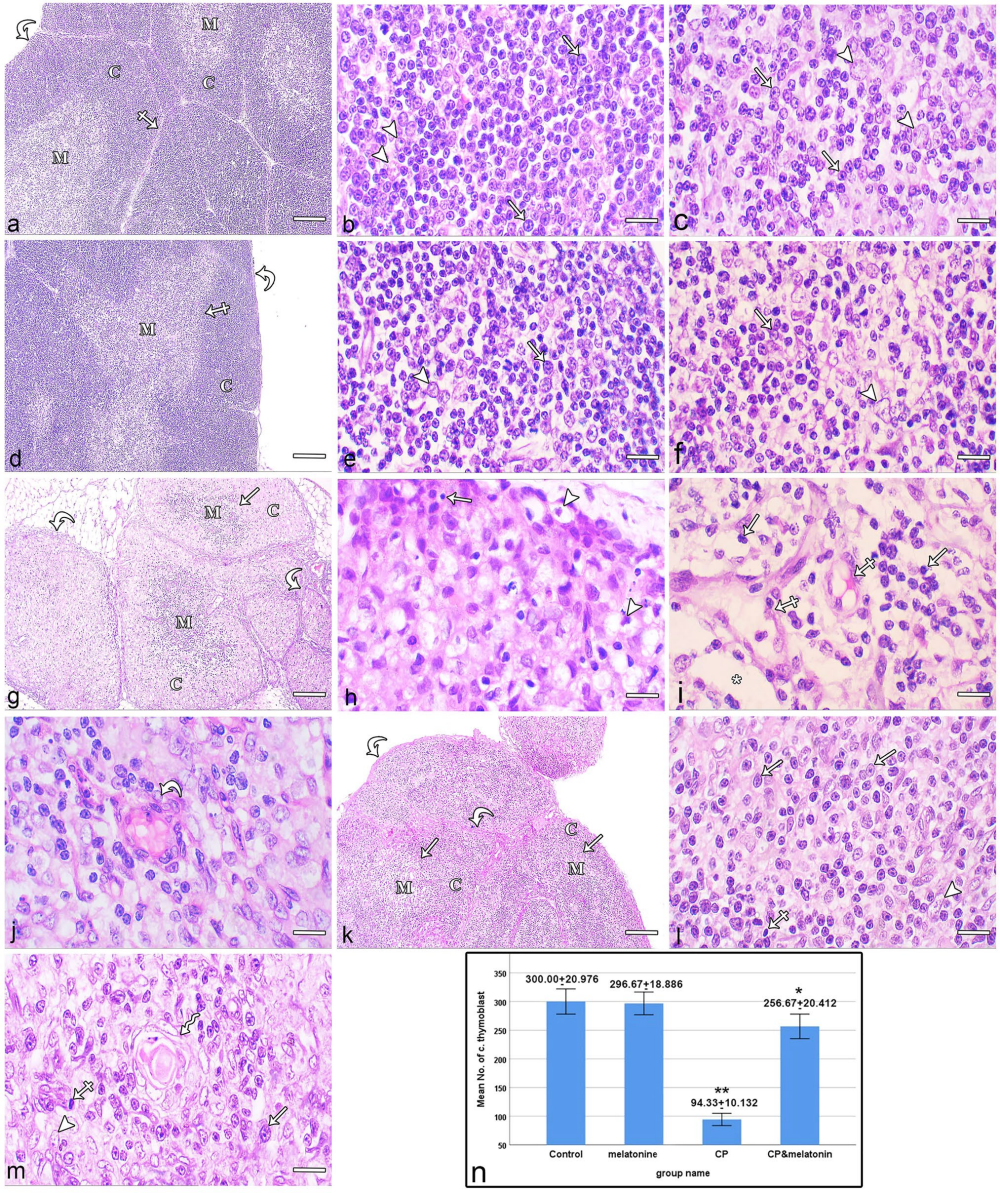
Photomicrographs of paraffin sections of thymus. (**a–c**) Control sections and (**d–f**) melatonin-treated sections. (**a**, **d**) The thymus is covered by a thin connective tissue capsule (curved arrows) and is divided into lobules by thin septa (crossed arrows). Each lobule is divided into an outer dark cortex (C) and an inner pale medulla (M). (**b**, **e**) The cortex contains thymoblasts with large round nuclei (arrows) and a few ERCs with oval pale-stained nuclei (arrowheads). (**c**, **f**) The medulla is composed of a small number of loosely packed thymocytes (arrows) and ERCs (arrowheads). (**g–j**) CP-treated sections. (**g**) An apparent marked thickening of the capsule and septa (curved arrows) is seen. The thymic lobules are involuted and inverted. The cortex (C) and medulla (M) thicknesses are clearly reduced, the boundary between them is not clear (arrow), and the medulla (M) appears darker than the cortex (C). (**h**) The cortex shows depletion of thymoblasts; most of them are shrunken, with condensed, darkly stained nuclei (arrow). The cortex is populated mainly with ERCs that show abundant vacuolated cytoplasm (arrowheads). (**i**) The medulla shows an apparent reduction in thymocyte numbers (arrows), leaving empty areas (*) and some dilated blood vessels (crossed arrows). (**j**) Enlarged Hassall’s corpuscle (curved arrow) can also be observed. (**k–m**) CP + melatonin-treated sections. (**k**) Thymus showing thin connective tissue capsule and septa (curved arrows). The thymic lobules show an outer dark cortex (C) and an inner pale medulla (M), with a distinct boundary in some lobules (arrow). (**l**) Cortex shows that most thymoblasts (arrows) and ERCs (arrowhead) are similar to those observed in the control. A few shrunken thymoblasts (crossed arrow) are also seen. (**m**) Medulla showing thymocytes (arrow) and ERCs (arrowhead) to be almost the same as the control. A few shrunken thymocytes are also seen (crossed arrow). Hassall's corpuscle (zigzag arrow) can also be observed. (**n**) Statistical analysis of the number of cortical thymoblasts (mean ± SD) in the thymus within different groups of the study. (**a**, **d**, **g**, **k**) Scale bars: 10 µm, (**b**, **c**, **e**, **f**, **h**, **i**, **j**, **l**, **m**) Scale bars: 100 µm

Examination of the thymic sections from the control group showed that the thymus gland was covered with a thin connective tissue capsule and divided into lobules by thin connective tissue septa. The lobules were composed of an outer darkly stained cortex and an inner pale medulla with a clear boundary between them (Fig. 1a). The thymic cortex contained many densely packed thymoblasts and a few epithelial reticular cells (ERCs). Thymoblasts exhibited large round nuclei. The ERCs contained oval to round pale-stained nuclei (Fig. 1b). The thymic medulla consisted of loosely packed thymocytes with small, rounded, deeply stained nuclei and more prominent ERCs (Fig. 1c). Examination of thymic sections from the melatonin-treated group revealed that the structure of the thymus was similar to that of the control group (Fig. 1d, e, f).

Examination of thymic sections of the CP-treated group revealed marked thickening of the connective tissue capsule and septa. The thymic lobules were involuted and inverted. The thickness of the cortex and medulla was markedly reduced, the boundary between them was not clear, and the medulla appeared darker than the cortex (Fig. 1g). The thymic cortex showed depletion of thymoblasts, most of which were shrunken and showed condensed, dark-stained nuclei. The cortex was populated mainly with ERCs that showed abundant vacuolated cytoplasm (Fig. 1h). In thymic medulla, an apparent decrease in the number of medullary thymocytes leaving empty areas could be observed. Most of the thymocytes were shrunken and had darkly stained nuclei. The ERCs were more prominent with oval pale-stained nuclei. In addition, Hassall’s corpuscles were enlarged and more frequently encountered than in the control group. They consisted of a central hyaline acidophilic mass surrounded by many ERC layers (Fig. 1i, j). Multiple dilated blood vessels were also observed (Fig. 1i).

Examination of thymic sections from the CP + melatonin-treated group showed preservation of thymic architecture. The thymus appeared more or less similar to the control. It was covered with a thin connective tissue capsule and divided by thin connective tissue septa. Each lobule exhibited an outer dark-stained cortex and an inner pale medulla (Fig. 1k). The thymic cortex showed that most thymoblasts and ERCs were more or less similar to those observed in the control group. A small number of shrunken thymoblasts was observed (Fig. 3l). The thymic medulla was apparently more or less similar to the control. It contained loosely packed thymocytes, more prominent ERCs, and Hassall's corpuscles. A few shrunken thymocytes could also be observed (Fig. 1m).

Compared with the control group, the mean number of cortical thymoblasts showed a nonsignificant decrease in the melatonin-treated group, a highly significant decrease (*P* < 0.001) in the CP-treated group and a significant decrease in the CP + melatonin-treated group. Moreover, the mean number of cortical thymoblasts in the CP + melatonin-treated group showed a highly significant increase (*P* < 0.001) compared with the CP-treated group (Fig. 1n).

### Immunohistochemically stained sections

**Fig. 2:**
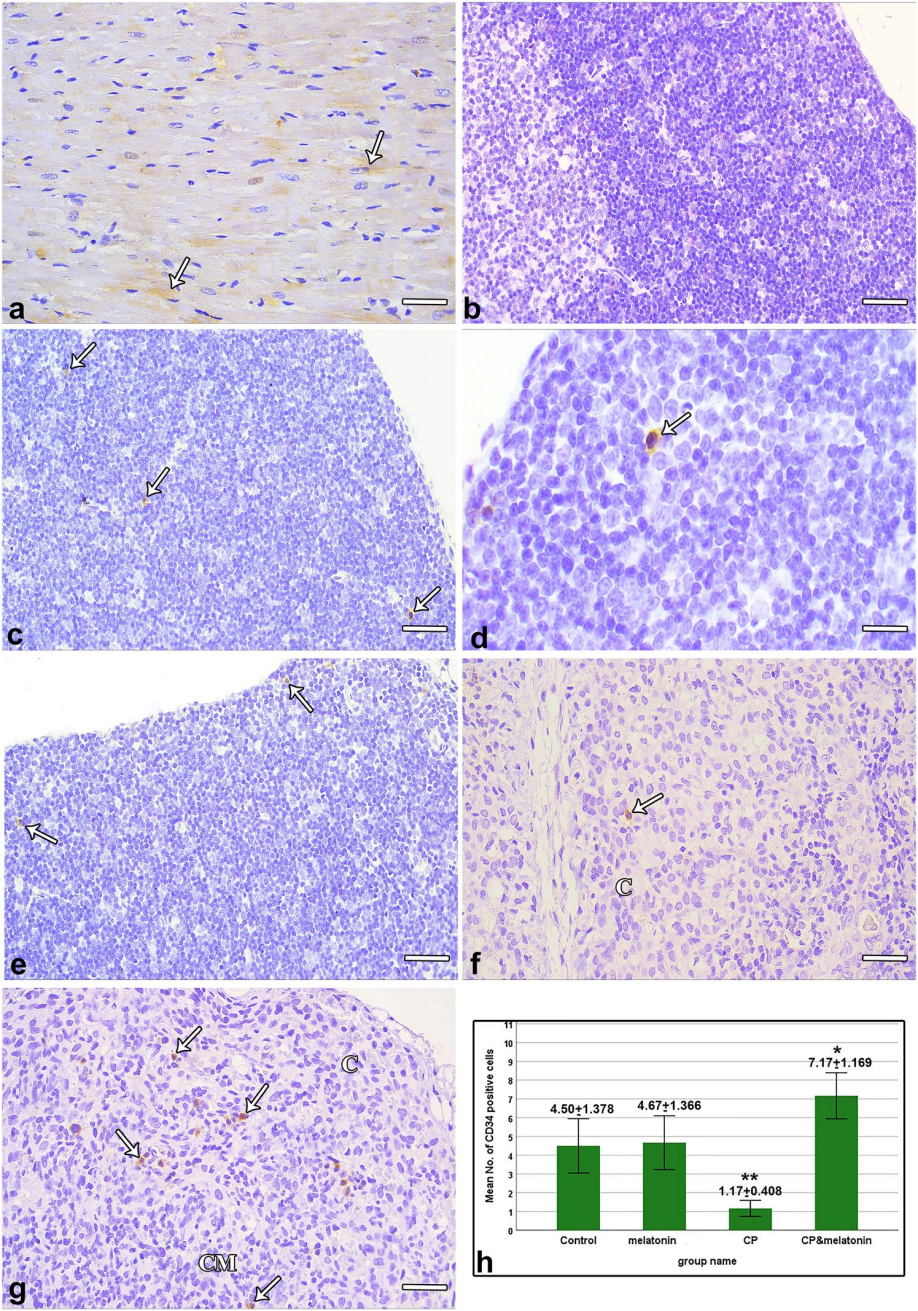
Photomicrographs of anti-CD34 immunostained sections. (**a**) Myocardial positive control showing brown positively stained cytoplasmic reaction to CD34 in cardiac stem cells (arrows). (**b**) Thymic negative control after omitting the primary Ab showing absence of the brown positively stained immune reaction. (**c–f**) Anti-CD34 immunostained thymic sections. (**c**, **d**) Control sections. (**c**) CD34 positively stained stem cells (arrows) present in the cortex. (**d**) A brown positive cytoplasmic immune reaction is seen (arrow). (**e**) Melatonin-treated sections contain CD34 positively stained stem cells (arrows) in the cortex. (**f**) CP-treated sections show one CD34 positively stained stem cell (arrow) in the cortical region (C). (**g**) CP + melatonin-treated sections contain numerous CD34 positively stained stem cells (arrows) distributed in the cortex (C) and corticomedullary junction (CM). (**h**) Statistical analysis of the number of CD34 positively stained stem cells in cortical regions of the thymus (mean ± SD) within different study groups. (**a–c**, **e–g**) Scale bars: 25 µm, (d) Scale bar: 100 µm

Examination of the myocardium (positive control) showed a brown positively stained cytoplasmic reaction for CD34 in cardiac stem cells (Fig. 2a). While examination of the thymic sections after omitting the primary Ab (negative control) showed no brown positively stained immune reaction (Fig. 2b).

Examination of thymus sections from the control group showed stem cells with a brown positive cytoplasmic immune reaction scattered throughout the thymus, especially in the cortex (Fig. 2c, d). The same immune reaction pattern was observed in the melatonin-treated group (Fig. 2e). Thymic sections from the CP-treated group occasionally showed CD34 positively stained stem cells (Fig. 2f). On the other hand, assessment of thymic sections from the CP + melatonin-treated group revealed numerous CD34 positively stained stem cells distributed in the cortex and corticomedullary junction (Fig. 2g).

Compared with the control group, the mean number of cortical CD34 immune positively stained stem cells showed a nonsignificant increase in the melatonin-treated group, a highly significant decrease (*P* < 0.001) in the CP-treated group, and a significant increase in the CP + melatonin-treated group (Fig. 2h).

### Toluidine blue-stained sections

**Fig. 3:**
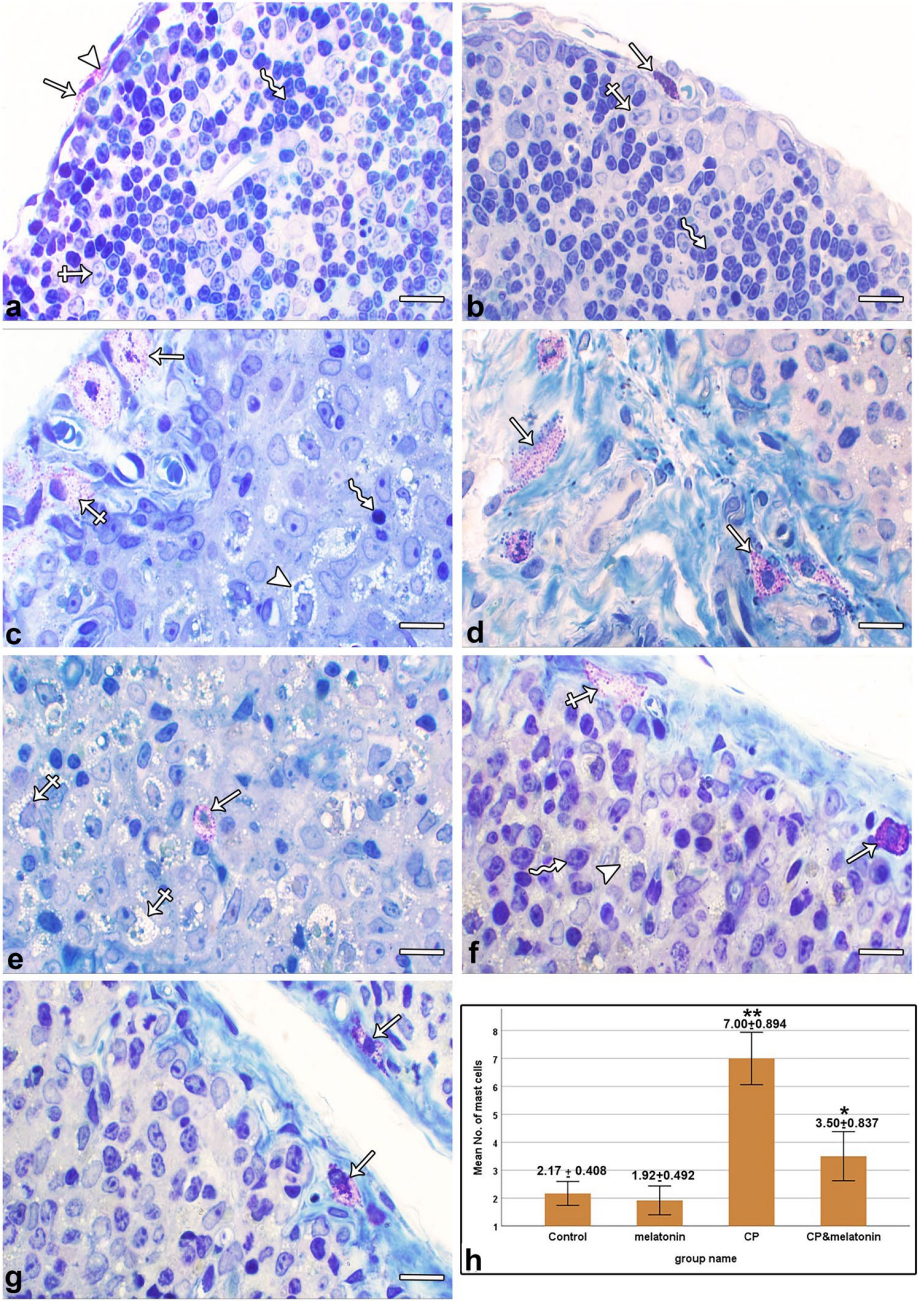
Photomicrographs of toluidine blue-stained semi-thin sections of the thymic cortex. (**a**) Control section shows a mast cell (arrow) within a connective tissue capsule. The mast cell contains numerous purple granules metachromatically stained with toluidine blue (arrowhead). The cortex is formed of numerous thymoblasts (zigzag arrow) and a small number of ERCs (crossed arrow). (**b**) The melatonin-treated section shows a mast cell in the connective tissue capsule. Thymoblasts (zigzag arrow) and ERCs (crossed arrow) are apparently similar to the control. (**c–e**) CP-treated sections. (**c**) An apparent marked increase in the number and size of partially degranulated mast cells in the thickened connective tissue capsule (arrows) and in the perivascular connective tissue (crossed arrow) can be seen. In addition, there is an apparent marked decrease in the number of thymoblasts (zigzag arrow) and ERCs appear more prominent with abundant vacuolated cytoplasm (arrowhead). (**d**) An apparent marked increase in the number and size of partially degranulated mast cells residing in the thickened interlobular septum (arrows). (**e**) One partially degranulated mast cell resides deep in the cortex of the thymus (arrow) and the cortex is populated mainly with ERCs that show abundant vacuolated cytoplasm (crossed arrows). (**f**, **g**) CP + melatonin-treated sections. (**f**) An apparent reduction can be seen in the number and size of mast cells within the connective tissue capsule as compared with group II. One mast cell is fully granulated (arrow) and the other is partially degranulated (crossed arrow). Thymoblasts (zigzag arrow) are more or less similar to the control. A small number of ERCs (arrowhead) show abundant vacuolated cytoplasm. (**g**) There is an apparent decrease in the number and size of partially degranulated mast cells (arrows) in the interlobular septum as compared with the CP-treated group. (**h**) Statistical analysis of thymic mast cell numbers (mean ± SD) in different study groups. Scale bars: 100 µm

Examination of semi-thin sections of the thymus of the control group showed almost no mast cells. Occasionally, mast cells with central nuclei were identified in the connective tissue capsule, interlobular septa, and perivascular connective tissue. Their cytoplasm contained numerous purple granules metachromatically stained with toluidine blue. The cortex consisted of a large number of densely packed thymoblasts and a small number of ERCs (Fig. 3a). Similar results were obtained in the melatonin-treated group (Fig. 3b).

Examination of thymic sections from the CP-treated group showed an apparent marked increase in the number and size of mast cells in the thickened connective tissue capsule, interlobular septa, and perivascular connective tissue. Some mast cells have been identified deep within the cortex of the thymus. Most of the mast cells appeared partially degranulated. In addition, the thymic cortex exhibited depletion of thymoblasts, was populated mainly with ERCs that showed abundant vacuolated cytoplasm (Fig. 3c, d, e).

On the other hand, examination of the thymic sections from the CP + melatonin-treated group showed an apparent decrease in the number and size of mast cells in the connective tissue capsule, interlobular septa, and perivascular connective tissue compared with group II. Some mast cells were fully granulated, others appeared to be partially degranulated. Most of the cortical thymoblasts and ERCs were apparently similar to the control group. A small number of ERCs that showed abundant vacuolated cytoplasm were still observed (Fig. 3f, g).

Compared with the control group, the mean number of mast cells showed a nonsignificant decrease in the melatonin-treated group and a highly significant increase (*P*<0.001) in the CP-treated group, with significant increases in the CP + melatonin treated group. Moreover, the mean number of mast cells in the CP + melatonin-treated group showed a highly significant (*P* < 0.001) decrease compared with the CP-treated group (Fig. 3h).

#### Electron microscopy results

**Fig. 4:**
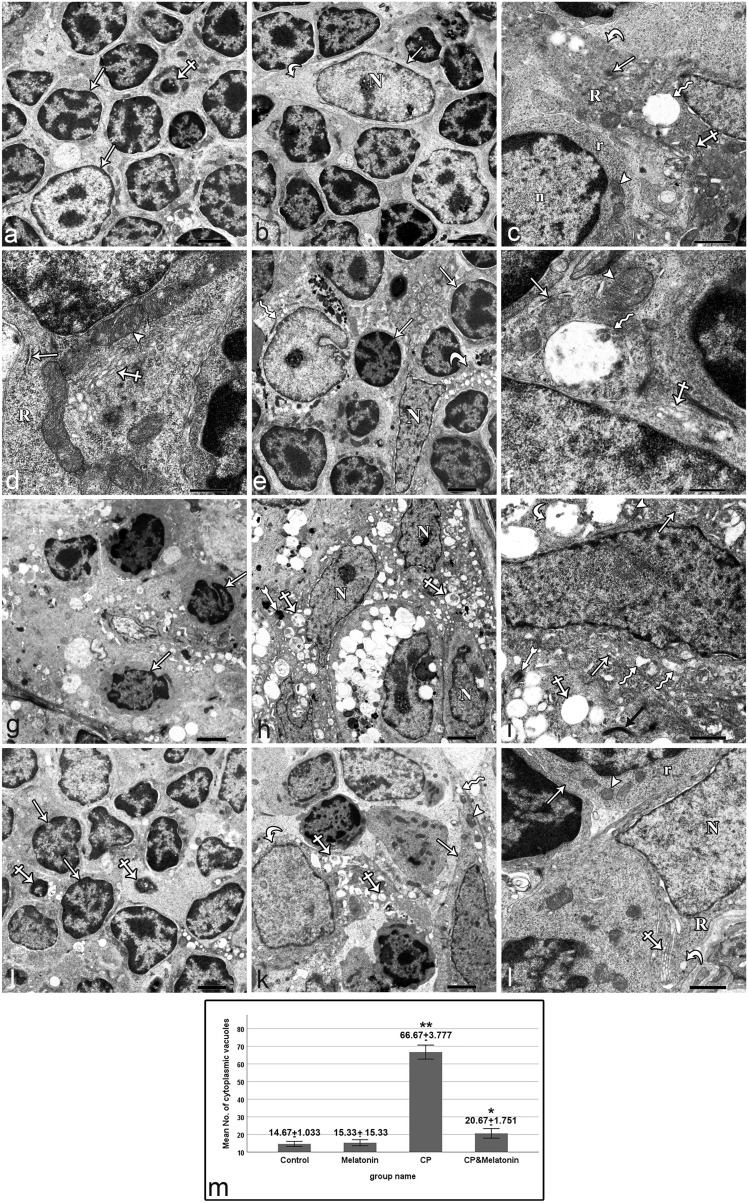
Electron micrograph of thymic cortex. (**a–d**) Control sections: (**a**) The cortex contains thymoblasts with heterochromatic nuclei (arrows). A pyknotic thymoblast can also be observed (crossed arrow). (**b**) A stellate-shaped ERC (arrow) shows a large euchromatic nucleus (N) and long cytoplasmic processes (curved arrow). (**c**) The ERC cytoplasmic process (curved arrow) contains Golgi apparatus (crossed arrow), free ribosomes (R), a few vacuoles (zigzag arrow) and tonofilaments (arrow). Part of the heterochromatic nucleus (n) of the thymoblast is also seen. Its cytoplasm contains mitochondria (arrowhead) and free ribosomes (r). (**d**) Mitochondria (arrowhead), rough endoplasmic reticulum (arrow), free ribosomes (R) and Golgi apparatus (crossed arrows) can also be seen the cytoplasm of one ERC. (**e**, **f**) Melatonin-treated sections: (**e**) The cortex contains thymoblasts with heterochromatic nuclei (arrows), a stellate-shaped ERC with a large euchromatic nucleus (N) and long cytoplasmic processes (curved arrow) and macrophage (zigzag arrow). (**f**) ERC cytoplasm contains mitochondria (arrowhead), rough endoplasmic reticulum (arrow), Golgi apparatus (crossed arrows) and few vacuoles (zigzag arrow). (**g–i**) CP-treated sections: (**g**) An apparent marked thymoblasts (arrows) depletion and degeneration are seen. (**h**) ERCs show irregular nuclei (N), containing numerous secondary lysosomes (bifid arrow) and numerous large vacuoles (crossed arrow) in the cytoplasm. (**i**) The cytoplasm of another ERC shows degenerated mitochondria (arrowhead), dilated rough endoplasmic reticulum cisternae (zigzag arrows), an apparent increase in the number of Golgi apparatus (arrows) and tonofilaments (bifid arrow). Numerous large vacuoles are also observed, some of which are electron-lucent (crossed arrow) and others appear with heterogeneous electron dense content (curved arrow). (**j–l**) CP + melatonin-treated sections. (**j**) Most thymoblasts appear apparently similar to the control (arrows). A few pyknotic thymoblasts (crossed arrows) can also be observed. (**k**) Two ERCs are seen. One of them is apparently similar to the control group (arrow), whose cytoplasm contains mitochondria (arrowhead) and a few vacuoles (zigzag arrow). The other ERC (curved arrow) shows numerous large vacuoles (crossed arrows). (**l**) ERC shows a euchromatic nucleus (N), whose cytoplasm contains Golgi apparatus (crossed arrow), free ribosomes (R) and a few vacuoles (curved arrow). Part of a thymoblast (arrow) can also be seen, the cytoplasm of which shows apparently normal mitochondria (arrowhead) and free ribosomes (r). (**m**) Statistical analysis of the number of cytoplasmic vacuoles (mean ± SD) in the ERCs of the thymic cortex within different study groups. (**a**, **b**, **e**, **g**, **h**, **j**, **k**) Scale bar: 2 µm, (**c**, **i**, **l**) Scale bar: 1 µm, (**d**, **f**) Scale bar: 500 nm

Examination of ultrathin thymus sections of the control group showed that the thymic cortex contained thymoblasts with heterochromatic nuclei occupying most of the cell. Their cytoplasm contained mitochondria and free ribosomes. In addition, we also observed a few pyknotic thymoblasts containing condensed nuclei and dark cytoplasm with barely visible organelles (Fig. 4a, c). ERCs appeared stellate-shaped with large euchromatic nuclei and long cytoplasmic processes. The cytoplasm of ERCs contained mitochondria with well-developed cristae, rough endoplasmic reticulum, free ribosomes, Golgi apparatus, a few vacuoles, and tonofilaments (Fig. 4b–d). Examination of the ultrathin thymus sections of the melatonin-treated group showed that the structure of the thymus was similar to that of the control group. The cortex contained thymoblasts with heterochromatic nuclei and stellate-shaped ERCs with a large euchromatic nuclei and long cytoplasmic processes. The ERC cytoplasm contained mitochondria with well-developed cristae, rough endoplasmic reticulum, Golgi apparatus, and a few vacuoles (Fig. 4e, f).

Examination of ultrathin sections of the CP-treated group revealed an apparent marked depletion and degeneration of the cortical thymoblasts. Most of them appeared shrunken with irregularly condensed nuclei and dark cytoplasm with barely visible organelles (apoptotic) (Fig. 4g). Most of the ERCs contained irregular nuclei, and their cytoplasm exhibited degenerated mitochondria, dilated rough endoplasmic reticulum cisternae, an apparent increase in the number of Golgi apparatus, more frequent secondary lysosomes, and numerous large vacuoles. Some of the vacuoles were electron-lucent and others appeared with heterogeneous moderate electron-dense content. Moreover, cytoplasmic tonofilaments were increased in quantity and electron density compared with the control group (Fig. 4h, i).

On the other hand, examination of ultrathin sections from the CP + melatonin-treated rat group showed an apparent protection of the thymic ultrastructure. Most of the thymoblasts were apparently similar to the control group. A few apoptotic thymoblasts were also observed (Fig. 4j, l). Most of the ERCs were apparently similar to the control group. They contained euchromatic nuclei, and the cytoplasm showed apparently normal mitochondria, Golgi apparatus, free ribosomes, and few vacuoles. A small number of other ERCs exhibited numerous large vacuoles with heterogeneous electron-dense content (Fig. 4k, l).

Compared with the control group, the mean number of cytoplasmic vacuoles in ERCs showed a nonsignificant increase in the melatonin-treated group and a highly significant increase (*P*<0.001) in the CP-treated group, with significant increases in the CP + melatonin-treated group. Moreover, the mean number of cytoplasmic vacuoles in ERCs in the CP + melatonin-treated group showed a highly significant (*P* < 0.001) decrease compared with the CP-treated group (Fig. 4m).

## Discussion

Cyclophosphamide (CP) is a widely used chemotherapeutic drug. Unfortunately, CP has been reported to induce thymic atrophy and increase the incidence of infection due to immunosuppressive alterations (Song et al. 2023). Melatonin is a potent antioxidant (Anghel et al. 2022).

The current work was performed to investigate the effects of CP on the histological architecture of the rat thymus. In addition, this study tried to assess the possible protective role of melatonin against CP-induced thymic changes.

In this study, rats of the CP-treated group received 200 mg/kg body weight CP by a single intraperitoneal injection, as it is a clinically relevant dose of cyclophosphamide chemotherapy. It is translated from the dosage given in breast cancer treatments and provides what may be a useful animal model (Janelsins et al. 2016).

CP-treated rats revealed marked histopathological changes in the thymus gland. The connective tissue capsule and septum were obviously thickened. This change in the connective tissue was attributed to CP-induced apoptosis of thymoblasts, which is compensated by connective tissue proliferation through CP-mediated activation of fibroblasts (Taha et al. 2015).

In the current study, light microscopic examination of the H&E-stained thymic sections from CP-treated rats revealed a depletion of cortical thymoblasts and an apparent reduction in medullary thymocyte numbers. These findings may be attributed to CP-induced oxidative stress and apoptosis (Cengiz et al. 2022). The chemotherapeutic functions of CP are mediated by its active metabolites, phosphoramide mustard, and the acrolein. Phosphoramide mustard, the ultimate alkylating agent, produces intra- and inter-strand DNA crosslinks or DNA–protein crosslinks and DNA strand breaks, inhibits DNA synthesis, and causes cell apoptosis. Acrolein metabolites produce cytotoxicity by inducing DNA single-strand breaks and play an important role in CP toxicity (Ayza et al. 2022).

An apparent increase in the size and the number of Hassall’s corpuscles was observed in CP-treated rats. Similar results have been reported by other authors (Haque and Khan 2017). The corpuscles appeared as dynamic structures associated with the structural changes in the thymus. The increase in the number and size of the corpuscles can be taken as evidence of thymic degeneration, which may be due to the role of Hassall's corpuscles in removing apoptotic thymoblasts (Rezaei 2020). It may also be due to various other cells that infiltrate Hassall's corpuscles during the degeneration (Haque and Khan 2017).

The vascular dilatation observed with CP treatment in our study may be due to CP-induced detachment of endothelial cells and disruption of their plasma membrane integrity (Abdelzaher et al. 2022; Krüger-Genge et al. 2018).

Examination of anti-CD34-stained thymus sections from CP-treated rats showed a statistically highly significant decrease. This decrease may be due to CP-induced myelosuppression of hematopoietic stem cell proliferation, which reduces stem cell production in the bone marrow and stem cell numbers in the thymus. In addition, CP has been reported to inhibit proliferation of all metabolically active cells, including stem cells (Yang et al. 2022).

In the current work, toluidine blue-stained semi-thin sections of the thymus of CP-treated rats showed a highly statistically significant increase in the number of mast cells compared with control rats. This increase can be attributed to CP-induced oxidative stress and cell damage, which can trigger an inflammatory response and increase the number of mast cells. In addition, ROS can induce mast cell degranulation (Kwack et al. 2022). Moreover, CP has been found to be able to increase the activity of nerve growth factor (NGF), a major promoter of mast cell migration, maturation, proliferation, and activation (Golubeva et al. 2014).

Mast cells have important immunological functions in normal conditions and in cases of thymic involution. Mast cell growth factor has been reported to stimulate stem cell proliferation and promote cortical thymoblast repopulation (Polevshchikov and Guselnikova 2021). Mast cell neuropeptides influence thymoblast maturation and differentiation. In addition, mast cell neuropeptides ensure attachment of proliferating thymoblasts to fibronectin in the thymic stroma. Mast cell-released substance P and neurokinin are critical for all stages of intrathymic T-cell development and their activation in peripheral lymphoid organs (Forsythe 2019).

In the current study, examination of ultrathin sections of thymus from CP-treated rats showed that most thymoblasts were apoptotic. ERCs also showed significant ultrastructural changes. Similar findings with CP treatment have been observed by other authors (Arudchelvan et al. 2005; Erokhina and Avilova 2019). These changes could be attributed to the cytotoxic effects of CP. Some authors reported that the numerous large vacuoles observed in ERCs with CP treatment could be secretory vacuoles, as the ERCs secrete biologically active factors that regulate thymoblast proliferation and maturation, and that the observed increased number of tonofilaments is associated with the exocytosis of these vacuoles. In addition, the vacuoles may be phagocytic vacuoles that engulf the dead thymoblasts and participate in the final breakdown and resorption of the destroyed cells (Arudchelvan et al. 2005).

Examination of the thymic sections of CP + melatonin-treated rats showed marked preservation of thymic architecture. This may be due to the antioxidant activity of melatonin, which acts directly as a free radical scavenger by detoxifying ROS and reactive nitrogen species, and indirectly by stimulating the activity of antioxidant enzymes and inhibiting the activity of pro-oxidants (Hagström et al. 2022).

The increase in the cortical CD34 positively stained stem cells in CP + melatonin-treated rats in this study was attributed to the melatonin-induced stimulation of migration and differentiation of hematopoietic stem cells to the thymus (Tutuncu and Delice 2021).

Toluidine blue-stained semi-thin sections from CP + melatonin-treated rats showed reduced mast cell numbers. Similar findings were observed by other authors with melatonin treatment (Çetin et al. 2020; Gaun et al. 2022). Nuclear factor kappa B (NF-κB) is a transcription factor that regulates the expression of proinflammatory cytokines from mast cells. Melatonin has been reported to inhibit NF-κB activation and translocation, downregulating mast cell activation, proliferation, and differentiation (Fusco et al. 2019).

## Conclusion

Cyclophosphamide induces structural and ultrastructural changes in the thymus. Melatonin may protect against CP-induced thymic injury.

## Supplementary Information

Below is the link to the electronic supplementary material.

Supplementary file1 (TIF 6718 KB)

Supplementary file2 (TIF 5512 KB)

Supplementary file3 (TIF 5515 KB)

Supplementary file4 (TIF 9229 KB)

Supplementary file5 (PDF 722 KB)
