# Supplementary figures and images for: Histological study of the role of CD34+ stem cells and mast cells in cyclophosphamide-induced thymic injury in rats and the possible attenuating role of melatonin
Source: Histochem Cell Biol. 2023 Mar 8;159(6):501–12. doi: 10.1007/s00418-023-02185-6 (PMC10247566; doi:10.1007/s00418-023-02185-6)

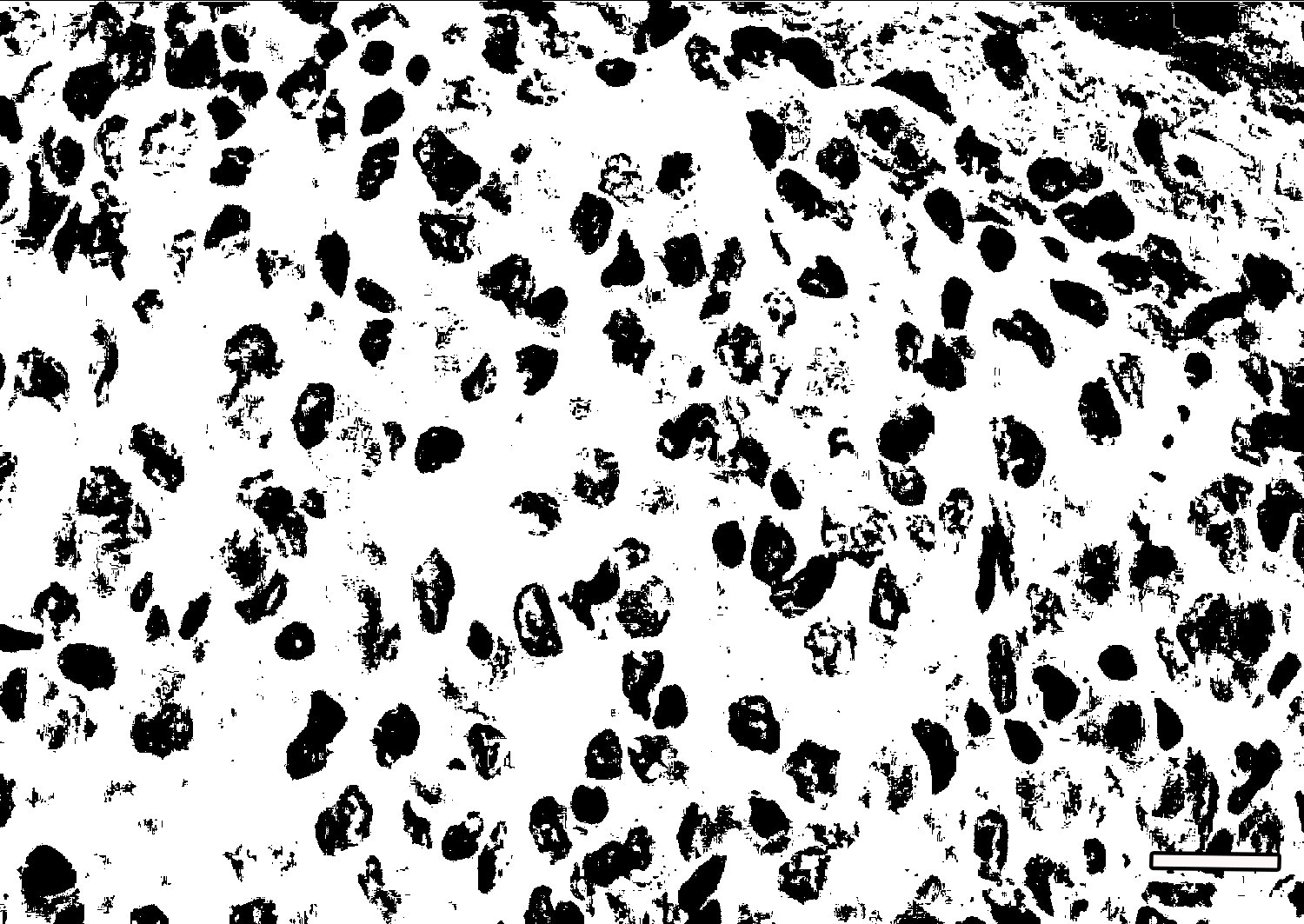

Supplement: Supplementary file 1 — Supplementary file1 (TIF 6718 KB) [file 418_2023_2185_MOESM1_ESM.tif]

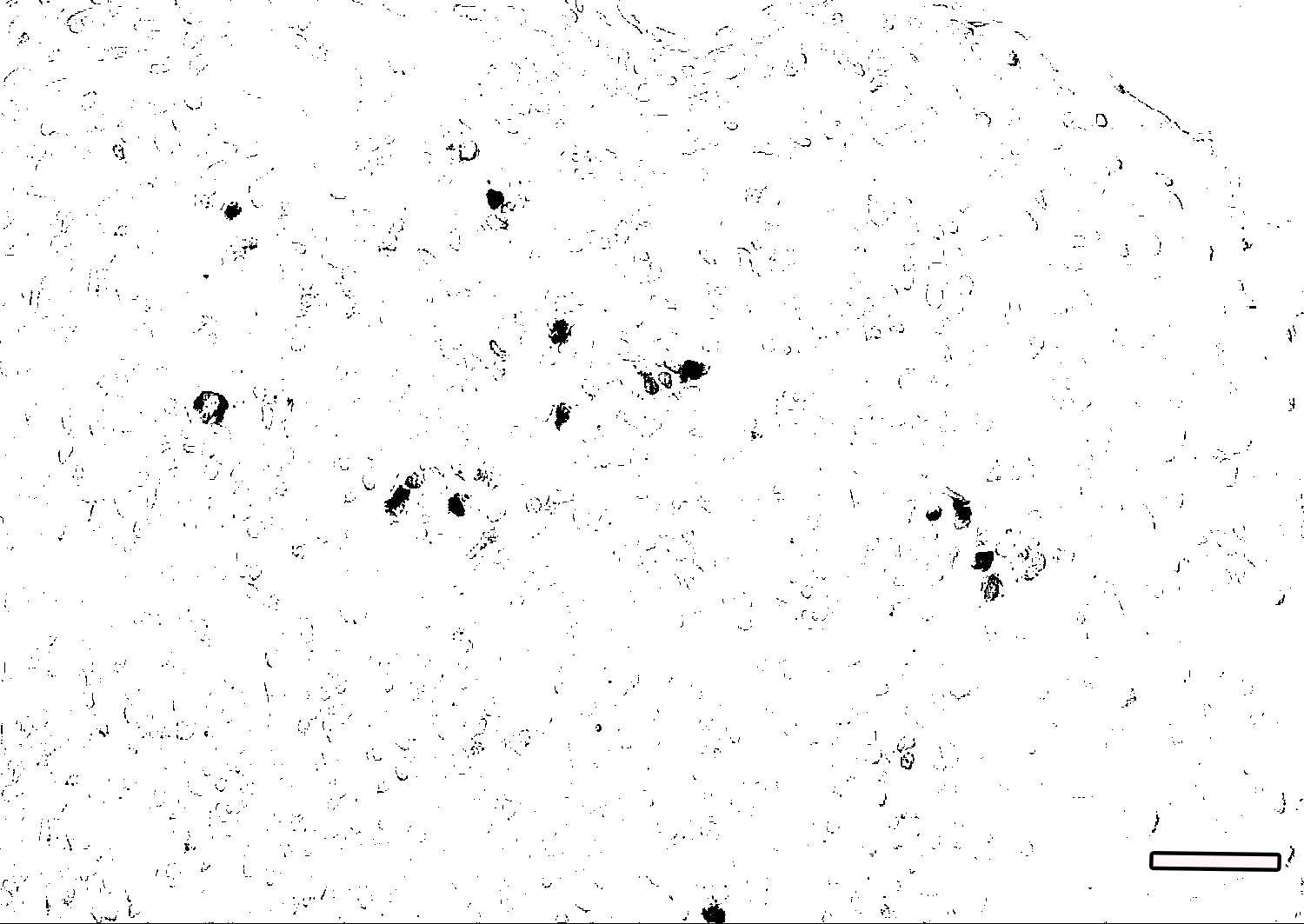

Supplement: Supplementary file 2 — Supplementary file2 (TIF 5512 KB) [file 418_2023_2185_MOESM2_ESM.tif]

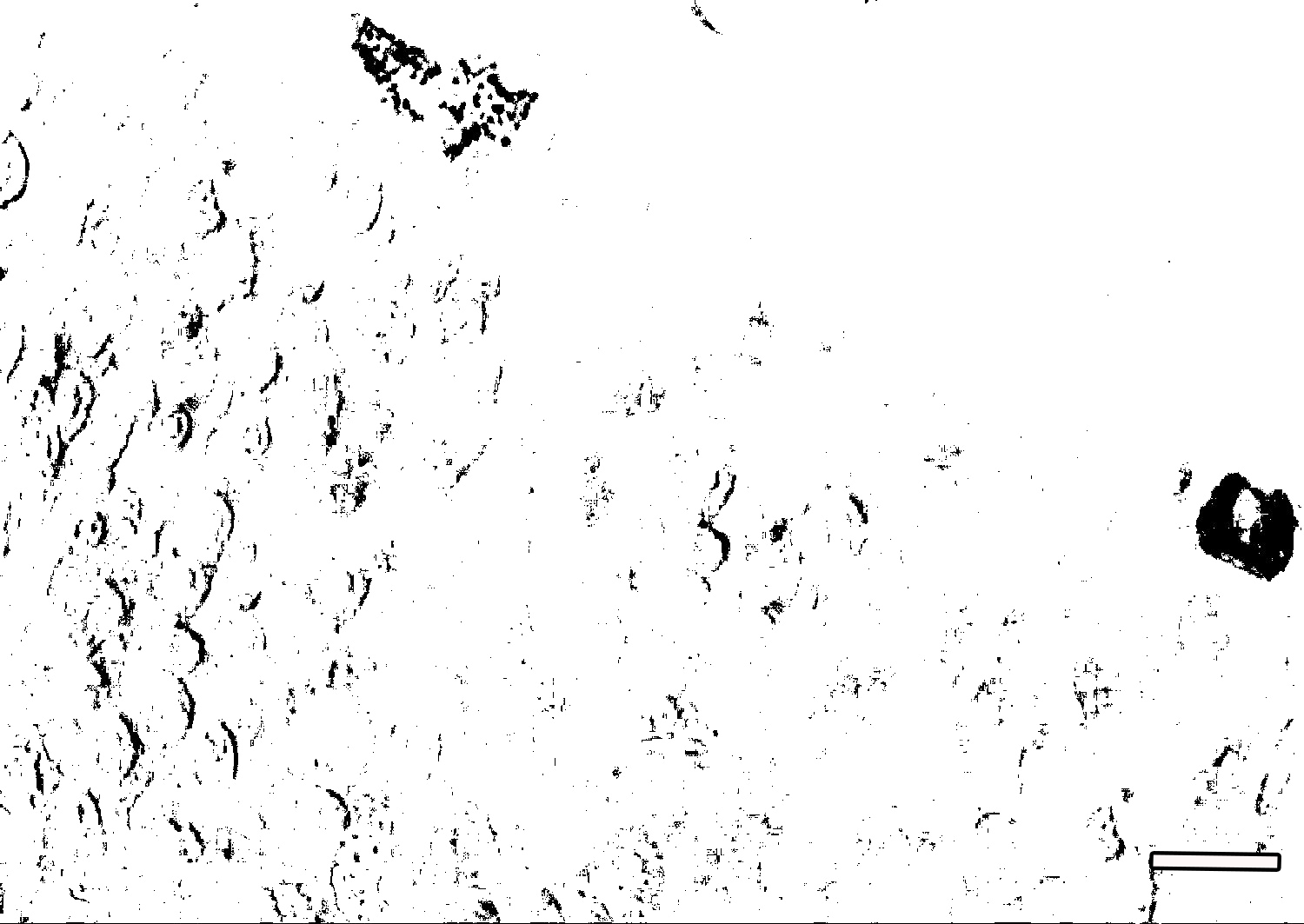

Supplement: Supplementary file 3 — Supplementary file3 (TIF 5515 KB) [file 418_2023_2185_MOESM3_ESM.tif]

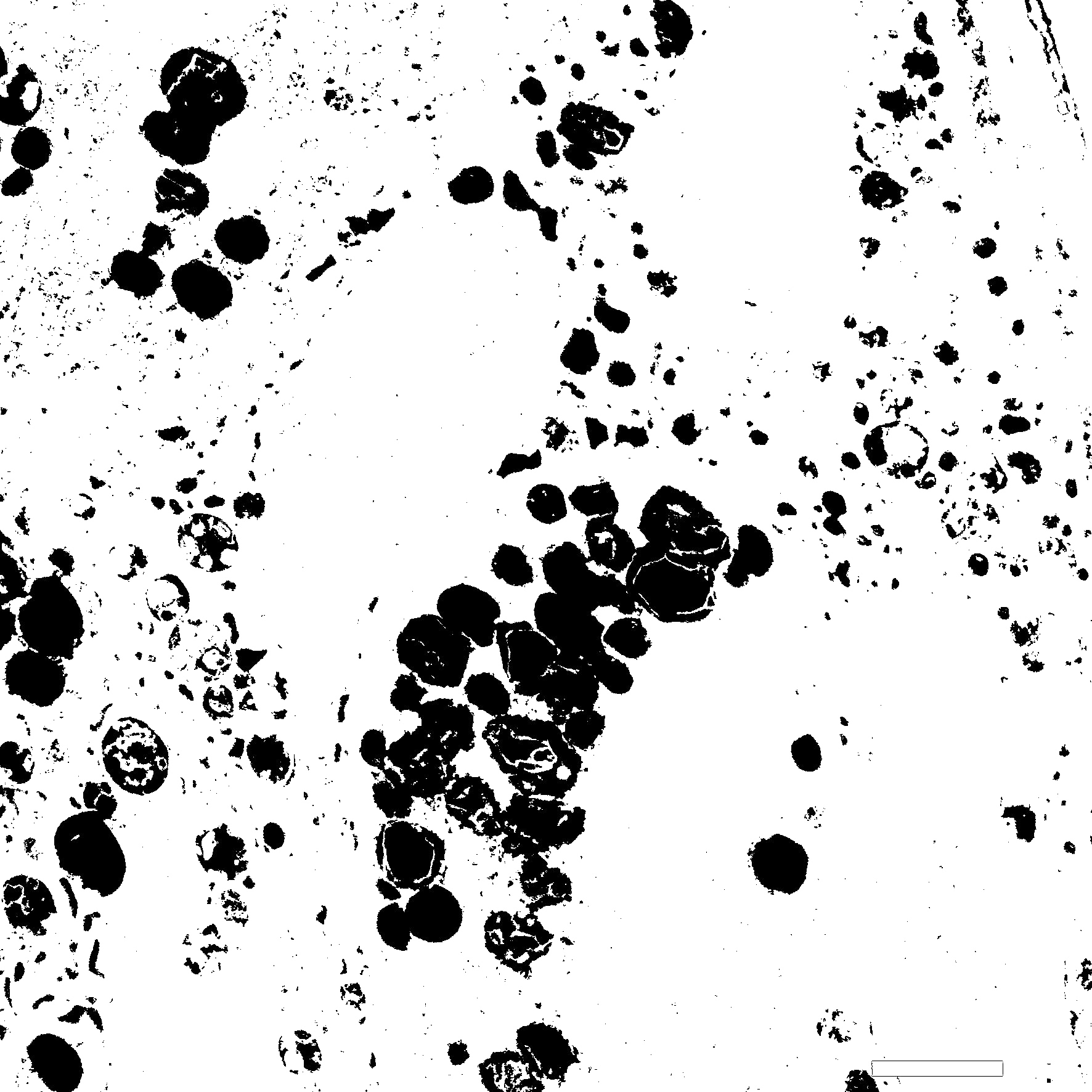

Supplement: Supplementary file 4 — Supplementary file4 (TIF 9229 KB) [file 418_2023_2185_MOESM4_ESM.tif]
