# Supplementary material for: Histological study of the role of CD34+ stem cells and mast cells in cyclophosphamide-induced thymic injury in rats and the possible attenuating role of melatonin
Source: Histochem Cell Biol. 2023 Mar 8;159(6):501–12. doi: 10.1007/s00418-023-02185-6 (PMC10247566; doi:10.1007/s00418-023-02185-6)
Supplement: Supplementary file 5 — Supplementary file5 (PDF 722 KB) [file 418_2023_2185_MOESM5_ESM.pdf]

Supplementary Figures

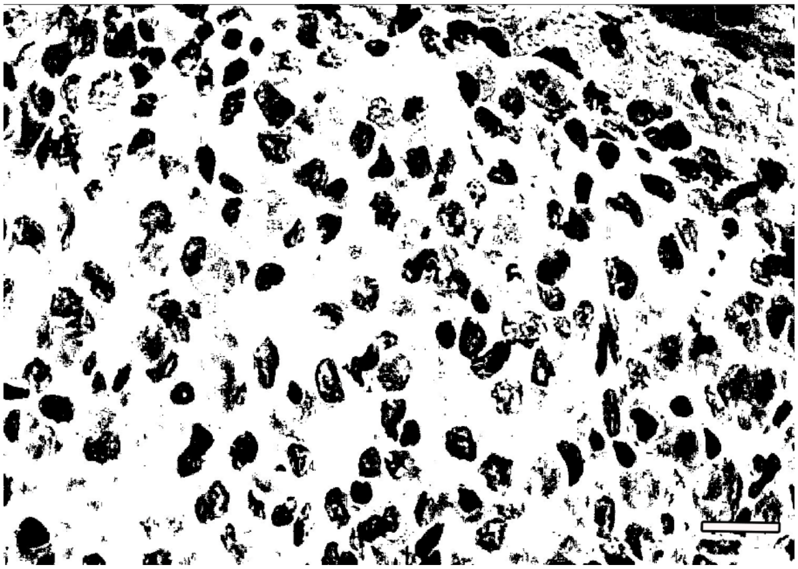

Fig. S1 Image assessing the number of cortical thymoblasts after adjustment of threshold using image J program. Scale bar: 100 µm.

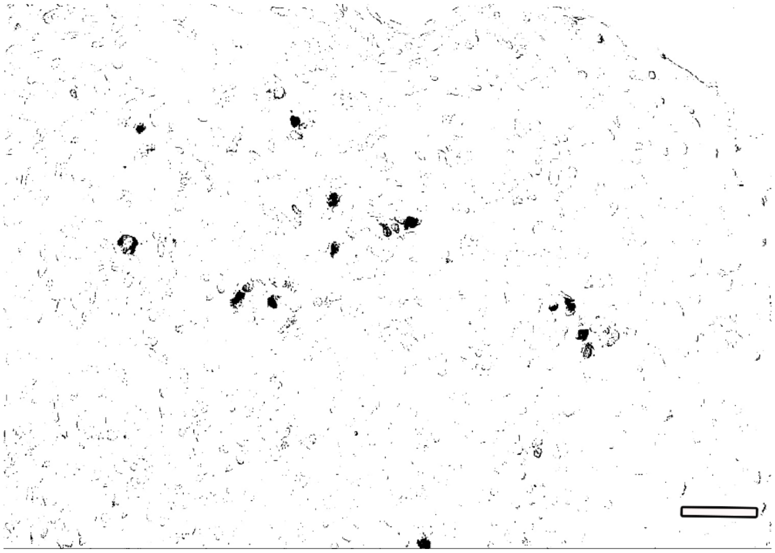

Fig. S2 Image assessing the number of cortical CD34 positively stained stem cells after adjustment of threshold using image J program. Scale bar: 25 µm.

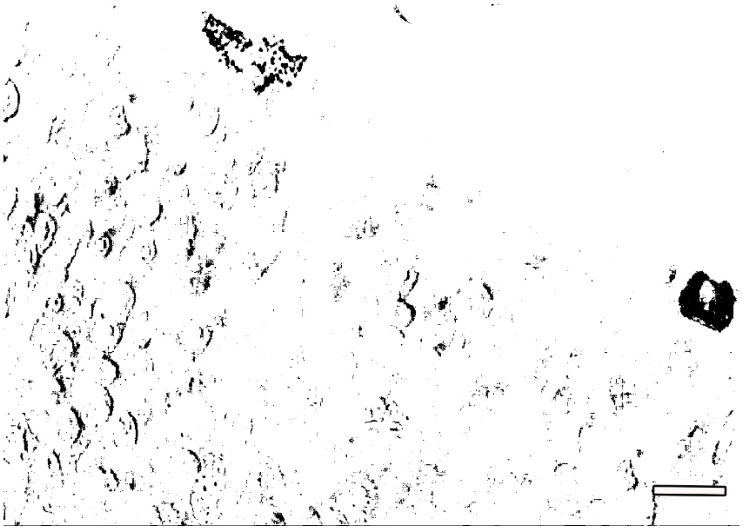

Fig. S3 Image assessing the number of mast cells after adjustment of threshold using image J program. Scale bar: 100 μm.

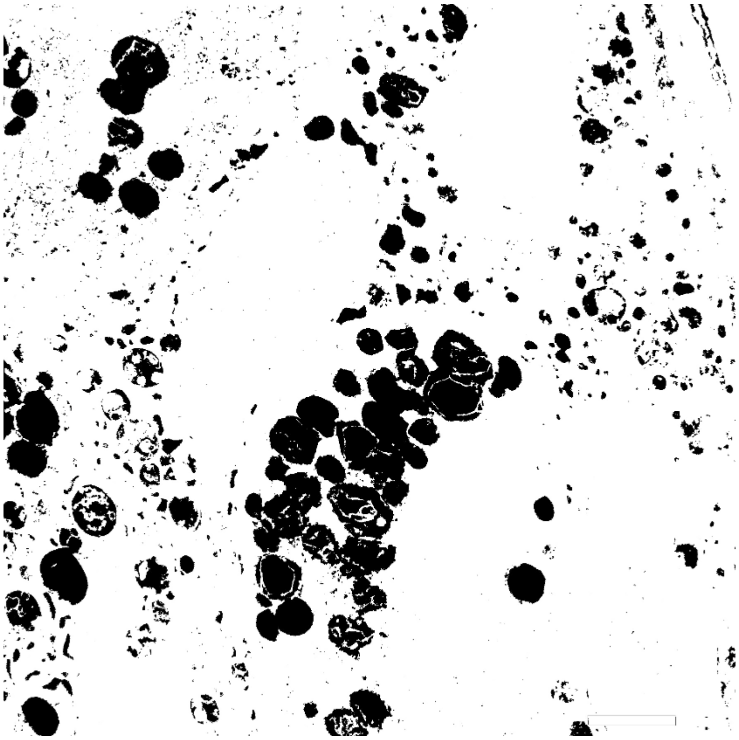

Fig. S4 Image assessing the number of cytoplasmic vacuoles in the ERCS after adjustment of threshold using image J program. Scale bar: 2 μm.
